# Drug safety: withdrawn medications are only part of the picture

**DOI:** 10.1186/s12916-016-0579-5

**Published:** 2016-02-13

**Authors:** Nigel S. B. Rawson

**Affiliations:** Eastlake Research Group, Oakville, ON Canada

**Keywords:** Drug safety, Adverse drug reaction, Drug withdrawal, Drug discontinuation

## Abstract

In a research article published in *BMC Medicine*, Onakpoya and colleagues provide a historical review of withdrawals of medications for safety reasons. However, withdrawn medications are only one part of the picture about how regulatory agencies manage drug risks. Moreover, medications introduced before the increased pre-marketing regulations and post-marketing monitoring systems instituted after the thalidomide tragedy have little relevance when considering the present drug safety picture because the circumstances under which they were introduced were completely different. To more fully understand drug safety management and regulatory agency actions, withdrawals should be evaluated within the setting and timeframe in which the medications are approved, which requires information about approvals and safety warnings. Studies are needed that provide a more comprehensive current picture of the identification and evaluation of drug safety risks as well as how regulatory agencies deal with them.

Please see related research article: http://bmcmedicine.biomedcentral.com/articles/10.1186/s12916-016-0553-2

## Background

Humanity has long sought to ease its sufferings with the use of medications, although few were effective and some were dangerous [1]. At the beginning of the 20th century, life expectancy was short, common infectious diseases had a high fatality rate and surgery was risky. However, many new medicines and vaccines were subsequently introduced that offered significant improvements in the quantity and quality of life. Some of the new medications had serious adverse effects, but the overall improvement in health up to 1960 generally overshadowed the risks.

The thalidomide disaster of 1961 [2] was a watershed in the history of drug safety that led to a major change in the understanding of and approach to drug safety. It also led to the establishment of drug monitoring agencies in many countries [3]. If an adverse drug reaction (ADR) outweighs a medication’s benefits, these agencies can require the product to be removed from the market.

## Review of drugs withdrawn for safety reasons

In a recent article in *BMC Medicine*, Onakpoya et al. [4] identified 462 medications discontinued for safety reasons in one or more countries between 1953 and 2013 and calculated three time periods for each product:

- ▪ Interval 1, years between launch year and year in which an ADR related to the reason for withdrawal was first reported;
- ▪ Interval 2, years between launch year and year of withdrawal; and
- ▪ Interval 3, years between year in which an ADR related to the reason for withdrawal was first reported and year of withdrawal.

They concluded that intervals 1 and 2 have shortened considerably over time, but there had been no consistent reduction in interval 3. However, using their data, all three intervals are seen to have decreased dramatically, indicating a significant improvement in identifying and acting upon major safety risks since the 1970s (Table 1).

**Table 1:** Intervals 1, 2 and 3 by launch period

Launch period	Interval	Number of medications	Median (years)	Interquartile range (years)
Pre-1961	1	187	20	9–32
	2	187	37	27–57
	3	187	16	6–28
1961–1975	1	128^a^	8	3–15
	2	131	17	10–22
	3	128^a^	5	1–11
1976–1990	1	75	3	2–5
	2	75	6	4–12
	3	75	2	0–6
1991–2013	1	69	1	0–4
	2	69	3	1–6
	3	69	1	0–2

^a^Year of the first ADR report related to the reason for withdrawal was missing for three medications

Although reviews of withdrawals are interesting from a historical perspective, medications introduced before the increased pre-marketing regulations and post-marketing monitoring systems instituted after thalidomide have little relevance when considering the current safety picture because the circumstances under which they were introduced were completely different. Over 40 % (187) of the medications in the review were introduced before 1961, with 64 (14 %) before the Second World War and 23 (5 %) before 1900, so that they include some that were brought into use when therapeutics consisted mainly of bleeding, purging, sweating and vomiting [1]. For instance, mercurous chloride (calomel) was prescribed by many American physicians for a variety of illnesses from the late 1700s to well into the 19th century, although its dangers were known before 1850 [5]. In addition, for 73 (16 %) of the medications, an ADR related to the reason for withdrawal was reported before 1961.

## Understanding the context of drug withdrawals

A review of withdrawals provides only part of the picture concerning medication safety and regulatory agency actions. The number of discontinued medications should be placed within the context of the overall number approved in a specific setting and timeframe so that withdrawal rates can be estimated. Information regarding drug approvals is publicly available for many developed countries and has been used to evaluate safety risks in these jurisdictions [6–11]. Similar information may be accessible for other countries, although language issues may present a problem. Information from less developed countries is likely to be more difficult to obtain. Nevertheless, pharmaceutical manufacturers can be approached for information about where their products are approved and pharmaceutical data companies may be able to provide information that would indicate where medications have been approved.

Although Onakpoya and colleagues acknowledge that the number of withdrawn medications represents ‘only a small fraction of overall approvals’ [4], such details are commonly ignored or overlooked by academics and politicians [12]. Therefore, it is critical that safety discontinuation rates are evaluated. Where they have been assessed in developed countries, the rate has remained steady and low at around 2 % since the 1960s, with a decline in recent years [6–11].

Among the withdrawn medications, 263 were approved after 1961, of which 40 % were discontinued in only one country. Since approval information is absent, it is unknown whether the medication was only approved in that country or whether it was approved in multiple jurisdictions and only one country required withdrawal. Each regulatory agency makes decisions regarding both marketing approval and drug safety issues based on priorities in its particular jurisdiction. Faced with reports of a serious ADR, some regulatory agencies may compel the manufacturer to include a major warning in the product details, while others may require the medication to be withdrawn. For example, rosiglitazone was withdrawn in Europe, New Zealand and other countries, but Australia, Canada and the United States have allowed it to remain on the market subject to stringent restrictions that severely limit its use [13–15].

Onakpoya et al. also report a lack of association between intervals 1 and 3 [4]. Both intervals have decreased, but there is no reason for the time taken to identify an ADR to be correlated with the period between first identification and discontinuation. Several years may elapse before a serious ADR is recognized, but it may take only days for the medication to be withdrawn; on the other hand, an ADR may be reported soon after a drug’s launch, but it may not be necessary to withdraw the medication quickly. Factors that can influence the duration of interval 1 are the source of the first ADR report (safety signals are more likely to be identified from reports to manufacturers under current pharmacovigilance practice, whereas case report publications were the main source of information in the past), the timing of the marketing application and its approval in each country (an ADR may be identified in the country where the medication is first submitted before other countries have reviewed the product), the size of the population (more rapid market penetration is likely to occur in large jurisdictions which increases the likelihood of early identification of an ADR), and physicians’ astuteness in recognizing an event as an ADR and their willingness to report it. Since not all risks are equal, the decision to withdraw a medication must take several considerations into account, such as whether the product has single or multiple indications, the availability of other therapeutic options and the regulatory agency’s approach to risk management, all of which can affect the extent of interval 3. The lack of information regarding approvals and safety warnings in each country means that a comprehensive picture of the relationship between identification and management of a safety issue cannot be constructed.

## Conclusions

Medications introduced and discontinued more than 25–30 years ago have little relevance to the present regulatory environment. Moreover, withdrawn medications are only one part of the picture of drug safety. Information about approvals and safety warnings by country is also required. Studies are needed that provide a holistic current picture of the identification and evaluation of drug safety risks as well as how regulatory agencies deal with them.
